# Johnson’s Operative Dentistry

**Published:** 1915-09

**Authors:** 


### BIBLIOGRAPHY

JOHNSON'S OPERATIVE DENTISTRY. The third edition of this work is just out, making three editions and five reprintings in as many years. This, probably, gives one as good a conception of the popularity of the work as is necessary to place an estimate on its worth. While the text is contributed by seventeen authors, the editorial work is that of Professor C. N. Johnson, of the Chicago Dental College. A new chapter on "The Treatment of Impacted Lower Third Molars," and "The Application of the Roentgen Ray to Dentistry," by Dr. E. C. Kells, makes a new subject for this, and, in fact, all such works. This subject should be treated in our oral surgical books, but it is so closely associated with other operative treatments that it fits into a book on operative dentistry appropriately The author's method is to anesthetize the pterygo-mandibular space by Fischer's conductive method, and then with a disk and burs cut off the impacted crown on its mesial surface, thus securing space to draw the root forward and up out of its socket. His claim is far less mutilation of the tissues involved and more satisfactory healing. The article is well written and illustrated and is a valuable addition to this technique.

The book shows more or less revisions in other chapters, but remains substantially unchanged in its general make up. It is well made, as are all the books of P. Blakiston's Son & Co.

